# Molecular landscape of HER2-mutated non-small cell lung cancer in Northeastern Brazil: Clinical, histopathological, and genomic insights

**DOI:** 10.18632/oncotarget.28737

**Published:** 2025-06-17

**Authors:** Cleto Dantas Nogueira, Samuel Frota, Huylmer Lucena Chaves, Juliana Cordeiro de Sousa, Guilherme de Sousa Veloso, Francisco Jonathan dos Santos Araujo, Gabriel Barbosa Silva, Samuel Silva Ferreira, Marclesson Santos Alves, Fabio Nasser, Ezequiel Rangel, Francisco Martins Neto, Iusta Caminha, Ellen Nascimento, Fabio Tavora

**Affiliations:** ^1^Postgraduate Program in Pathology, Federal University of Ceará, Fortaleza, Brazil; ^2^Argos Pathology Laboratory, Fortaleza, Brazil; ^3^Postgraduate Program in Natural Resources Biotechnology, Center for Agricultural Sciences, Federal University of Ceará, Fortaleza, Brazil; ^4^Department of Pathology, Messejana Heart and Lung Hospital, Fortaleza, Brazil; ^5^InCor-HCFMUSP, São Paulo, Brazil; ^6^Thoracic Division, São Carlos Imagem Hospital, Fortaleza, Brazil; ^7^Institute D'Or for Research and Education, Fortaleza, Brazil; ^*^These authors contributed equally to this work

**Keywords:** HER2 mutation, NSCLC, lung cancer, targeted therapy, genomic profiling

## Abstract

HER2 genomic alterations characterize a specific subset of NSCLC with potential therapeutic relevance. While most studies focus on populations from high-income countries, data from Latin America remains scarce. We retrospectively analyzed 13 HER2-mutated NSCLC cases from a single institution in Northeastern Brazil, integrating clinical, histopathological, immunohistochemical, and molecular findings. Predominant histological patterns included acinar and lepidic subtypes, with HER2 mutations primarily involving exon 20 insertions (A775_G776insYVMA) and frequent co-alterations in TP53, KRAS, and STK11. HER2 protein expression assessed by IHC showed low scores (0–2+) in most cases, while HER2 gene amplification was confirmed in one case by D-DISH and NGS. Tumor mutation burden was universally low. Treatment responses varied, with one patient receiving trastuzumab deruxtecan. Our findings highlight the molecular diversity and diagnostic challenges of HER2-mutated NSCLC in underrepresented populations, emphasizing the need for comprehensive molecular profiling and expanded access to targeted therapies.

## INTRODUCTION

Dysregulation of HER2 is a key mechanism in the pathogenesis of breast and gastric cancers [1]. HER2 gene amplification occurs in approximately 15 to 20% of breast carcinomas and is responsible for protein overexpression in about 90% of these tumors [2, 3]. Additionally, it serves as a predictive marker for treatment with HER2-targeting agents [2]. Alterations in HER2, including overexpression, amplification, and/or activating mutations, have also been observed in other neoplasms, such as those of the ovary, endometrium, bladder, colon, biliary tract, and lung [4].

In non–small cell lung cancer (NSCLC), alterations in the HER2 (ERBB2) gene define a unique molecular subtype. HER2 abnormalities stem from gene mutation, amplification, or protein overexpression, with overexpression found in 10–15% of NSCLCs and up to 30% of lung adenocarcinomas. HER2 mutations occur in 2–4% of cases, and amplifications in 1–3% [5, 6] Mutations in the HER2 (ERBB2) gene define a distinct molecular subtype of lung cancer, primarily found in adenocarcinomas, with higher prevalence among women and non-smokers. Most of these mutations occur in the tyrosine kinase domain, with approximately 90% consisting of insertions in exon 20 [7].

Trastuzumab deruxtecan (T-DXd) is the first HER2-targeted agent to show clinical efficacy in HER2-mutant non-small cell lung cancer (HER2m NSCLC). It has been approved in multiple countries, including accelerated FDA approval in the U.S., for patients with unresectable or previously treated HER2m NSCLC [6]. More recently, the FDA expanded its indication to include unresectable or metastatic solid tumors across various primary sites with HER2 overexpression (immunohistochemistry score 3) [8, 9]. These approvals require the use of a companion diagnostic assay to identify eligible patients. Accurate detection of HER2 mutations or overexpression is critical for optimizing the therapeutic potential of T-DXd [8, 9].

The complexity of genotypic and proteomic alterations in HER2 tumors has garnered significant interest, particularly in defining predictive biomarkers to guide patient selection for recently approved or in-development therapies, maximizing treatment strategies, and understanding mechanisms of resistance to targeted therapies. Despite the growing body of research on NSCLC harboring HER2 alterations, there is a notable lack of data on how these alterations present in Latin American populations. In this study, we analyzed a small cohort of patients from Northeast Brazil with HER2-mutant non-small cell lung cancer, conducted a comprehensive evaluation of HER2-associated alterations, and discussed the challenges in achieving accurate diagnosis.

## RESULTS

### Study population and clinical findings

**Figure 1:**
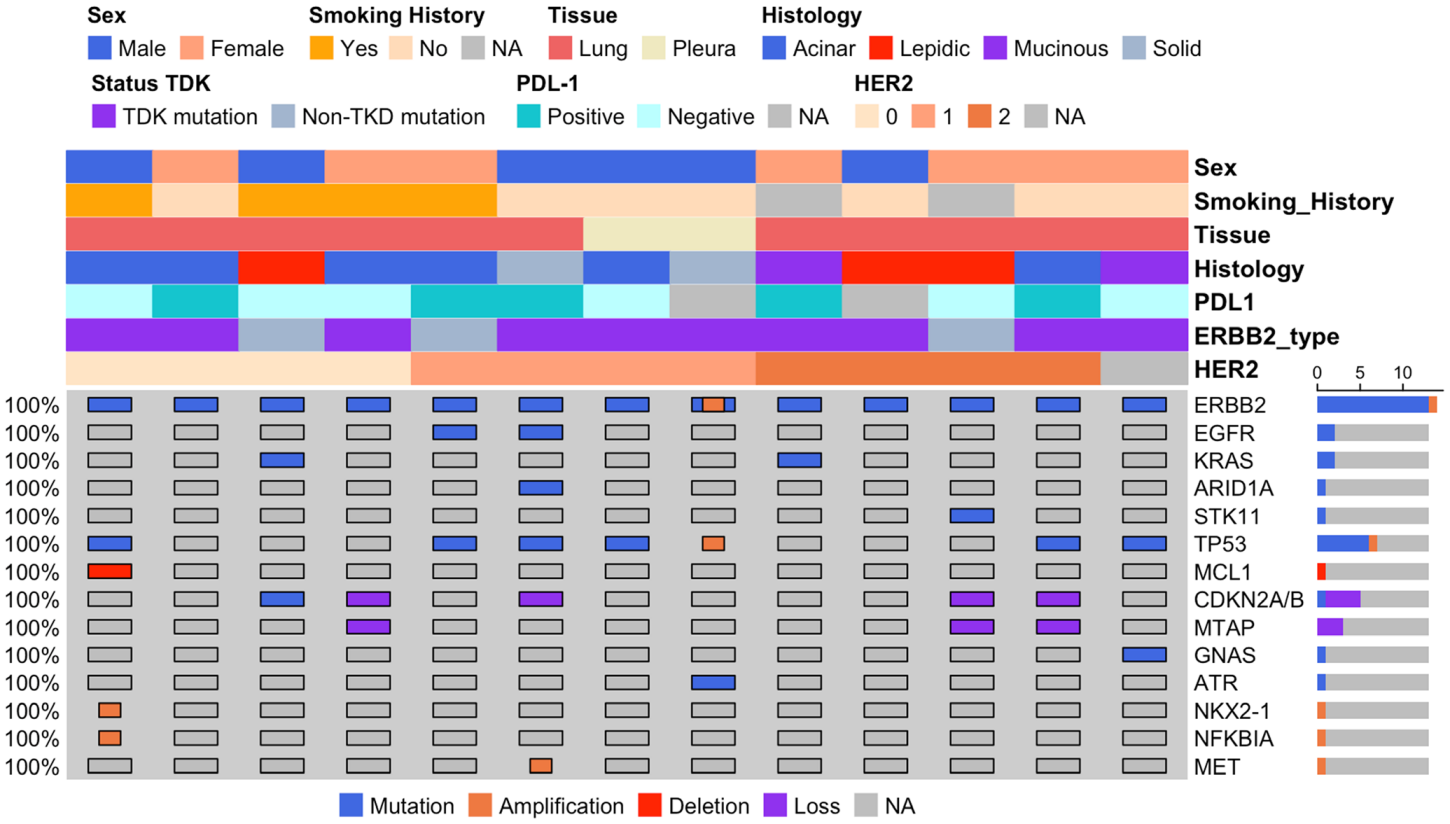
Clinical, histological, and molecular features of 13 lung cancer cases with HER2 alterations. The heatmap summarizes the clinical, histological, and molecular profile of 13 lung cancer patients with HER2 mutations. Key characteristics, including sex, smoking history, tissue origin (lung or pleura), histological subtype (acinar, lepidic, mucinous, or solid), HER2 status by IHC (0, 1+, 2+, or NA), PD-L1 status (positive, negative, or NA), and type of HER2 mutation (TKD or non-TKD), are depicted. The lower panel displays additional genomic alterations, including mutations, amplifications, deletions, and losses in frequently altered genes (e.g., ERBB2, EGFR, KRAS, TP53). The bar plot to the right indicates the frequency of alterations across the cohort. This comprehensive profile highlights the heterogeneity in clinical, immunohistochemical, and genomic features in HER2-mutated lung cancer cases.

**Figure 2:**
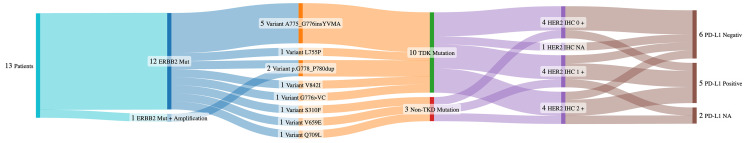
Distribution of HER2 mutations, HER2 IHC expression, and PD-L1 status in a cohort of 13 lung cancer patients. Sankey diagram illustrating the molecular and immunohistochemical landscape of HER2-mutated lung adenocarcinomas. This diagram visualizes the classification pathway of 13 lung cancer patients harboring HER2 alterations, highlighting the distribution and relationships among HER2 mutation variants, mutation domain (TKD vs. non-TKD), HER2 protein expression by immunohistochemistry (IHC), and PD-L1 expression status. The flow begins with 13 patients, of whom 12 harbored HER2 mutations alone and one presented with both HER2 mutation and amplification. Specific HER2 variants are shown, with the most common being A775_G776insYVMA. These variants are subsequently grouped by mutation location: tyrosine kinase domain (TKD) mutations (*n* = 10) and non-TKD mutations (*n* = 3). The next level displays HER2 IHC results (0, 1+, 2+, or not available (NA)), followed by PD-L1 status (positive, negative, or NA). The diagram highlights the heterogeneity of HER2-mutant tumors in terms of both protein expression and immune marker status. For instance, HER2 IHC scores were not consistently correlated with mutation type or PD-L1 expression. This comprehensive view underscores the complexity of biomarker interplay in HER2-altered non-small cell lung cancer (NSCLC) and supports the need for multi-modal molecular profiling in therapeutic decision-making. The width of each flow is proportional to the number of patients it represents. Each pathway from left to right shows how patients are distributed across molecular subtypes and immunohistochemical profiles.

A total of 13 cases were identified among 263 cases of NSCLC diagnosed in a reference laboratory in Northeastern Brazil with NGS data. Their clinical and pathological characteristics are summarized in Table 1. The cohort had a wide age range, from 34 to 82 years, with a median age of 58. Females represented the majority of the cohort (54%, *n* = 7). Smoking history was diverse; 46% of the patients were never smokers, while the rest reported a history of smoking, ranging from light (5 pack-years) to moderate-heavy (40 pack-years). For three patients, smoking status was unknown. Tumor sizes ranged from 1.8 cm to 5.8 cm, with clinical stages spanning from IA1 to IVB. Early-stage tumors (stage I) accounted for 31% of the cases, while advanced stages (IVA and IVB) represented the majority, with over 50% of the patients presenting with stage IV disease. Most biopsies were performed on lung tissue, with pleural biopsies noted in two cases. PD-L1 expression showed a range from 0 to 90% of tumor cells, with most cases being negative (TPS 0). Figures 1 and 2 show clinical, histological and molecular features of the cases.

**Table 1:** Clinical and pathological characteristics of patients with HER2-mutated lung tumors

Patient	Age	Sex	Smoking history (pack-years)	Nodule size on CT	Clinical Stage	Biopsy on diagnosis	PD-L1	Rx	Follow-up	Follow-up in days
1	58	F	Unknown	2,0 cm	IB	Lung	10	Surgery	Alive and Well	121
2	39	M	Never	2,5 × 1,3 cm	IA1	Lung	Unknown	Surgery	ALWD	146
3	77	F	25	5,8 × 5,3 × 3,8 cm	IIB	Lung	10	EGFR TKI	ALWD	314
4	58	M	Never	0,7 × 0,1 cm	IVB	Lung	90	Immunotherapy and radiation in oligometastatic disease	ALWD	381
5	37	F	Unknown	2,2 cm	IVA	Lung	0	Systemic palliative chemo	DOD	40
6	78	M	Never	3,0 × 2,8 × 0,9 cm	IVA	Pleura	0	T-Dx + IO	DOD	780
7	58	M	5 – quit 30 years prior		IVB	Lung	0	Conventional chemo	DOD	466
8	82	F	Never	3,6 × 3,4 cm	IIA	Lung	0	Surgery	ALWD	817
9	51	F	Never	1,8 × 1,2 cm	IVB	Lung	1	Chemo + IO	ALWD	155
10	73	M	12	5,6 × 3,2 cm	IVB	Lung	70	Surgery and adjuvant chemo	Alive and Well	2167
11	72	F	15	1,8 × 1,5 cm	IVB	Lung	0	Chemo + IO	DOD	410
12	34	F	Never	1,2 × 0,2 cm.	IVA	Lung	0	Chemo followed by trastuzumab	DOD	734
13	53	M	40	3,7 × 3,2 × 0,4 cm	IVA	Pleura	0	No therapy - palliative	DOD	91

Abbreviations: CT: Computed Tomography; RX: Treatment; F: Female; M: Masculine; PD-L1: Programmed-Death Ligand 1; TKI: Tyrosine Kinase Inhibitors ALWD: Alive with Disease; DOD: Dead of Disease; IO: Immunotherapy; T-Dx Drug Conjugate Trastuzumab Deruxtecan.

### Treatment modalities

Treatment modalities varied significantly depending on disease stage and patient characteristics. Early-stage patients underwent surgical resection, often with curative intent, whereas advanced-stage patients received systemic therapies. These therapies included targeted treatments such as trastuzumab-deruxtecan (T-Dx) in only one patient, immunotherapy (IO), chemotherapy, and a combination of these modalities. One patient with oligometastatic disease was treated with radiation and immunotherapy, showcasing a multidisciplinary approach. Follow-up data revealed heterogeneous outcomes. At the time of the last follow-up, four patients were alive and well, with survival times ranging from 121 to 2167 days. Unfortunately, eight patients succumbed to their disease, with follow-up durations varying from 40 to 734 days. One patient was alive with disease at 817 days. These findings highlight the variability in clinical progression and outcomes among HER2-mutated lung cancer patients.

### Pathologic and molecular findings

Table 2 summarizes the molecular and histological characteristics of the 13 HER2-mutated lung tumors. The predominant histological subtypes included acinar (46%, *n* = 6), lepidic (23%, *n* = 3), solid (15%, *n* = 2), and mucinous (15%, *n* = 2).

**Table 2:** Molecular and histological characteristics of HER2-mutated lung tumors

Patient	Predominant histology	HER-2 Mutation	IHC HER-2	D-DISH HER-2 ratio	Other mutations	Tumor mutation burden	NGS platform
1	Mucinous	ERBB2 V842I	2	2,8	KRAS G12V/V14I	^*^	ONCOMINE
2	Lepidic	ERBB2 L755P	2	1,9	^*^	^*^	ONCOMINE
3	Acinar	ERBB2 S310F	1	2,8	EGFR L858R TP53 R213^*^	^*^	ONCOMINE
4	Solid	ERBB2 G776>VC	1	3,7	EGFR F376Y TP53 N210fs^*^6 ARID1A Q611^*^ CDKN2Ab loss	2 mut/Mb	FOUNDATION ONE
5	Lepidic	ERBB2 V659E	2	2,4	STK11 921-1G>T	1 mut/Mb	FOUNDATION ONE
6	Acinar	ERBB2 A775_G776insYVMA	1	1,3	TP53 D42fs^*^2	1 mut/Mb	FOUNDATION ONE
7	Acinar	ERBB2 A775_G776insYVMA	0	2,0	TP53 Y220C MCL1 E110del	3 muts/Mb	FOUNDATION ONE
8	Acinar	ERBB2 A775_G776insYVMA	2	2,2	TP53 S240R	4 muts/Mb	FOUNDATION ONE
9	Acinar	ERBB2 G778_P780dup	0	2,0	^*^	^*^	ONCOMINE
10	Lepidic	ERBB2_Q709L	0	2,2	KRAS G12C CDK2NAb loss	5 muts/Mb	FOUNDATION ONE
11	Acinar	ERBB2 A775_G776insYVMA	0	2,1	MTAP Loss CDKN2A/B Loss	1 mut/Mb	FOUNDATION ONE
12	Mucinous	ERBB2 A775_G776insYVMA	?	2,4	TP53 R175H GNAS R201S	Unconclusive	FOUNDATION ONE
13	Solid	ERBB2 G778_P780dup	1	4,0	TP53 Q165 ATR c.2342-2A>C	13 muts/Mb	FOUNDATION ONE

Abbreviation: IHC: Immunohistochemistry; D-DISH: Dual-Color Dual in Situ Hybridization; NGS: Next Generation Sequencing; mut/Mb: mutations per megabase.

**Figure 3:**
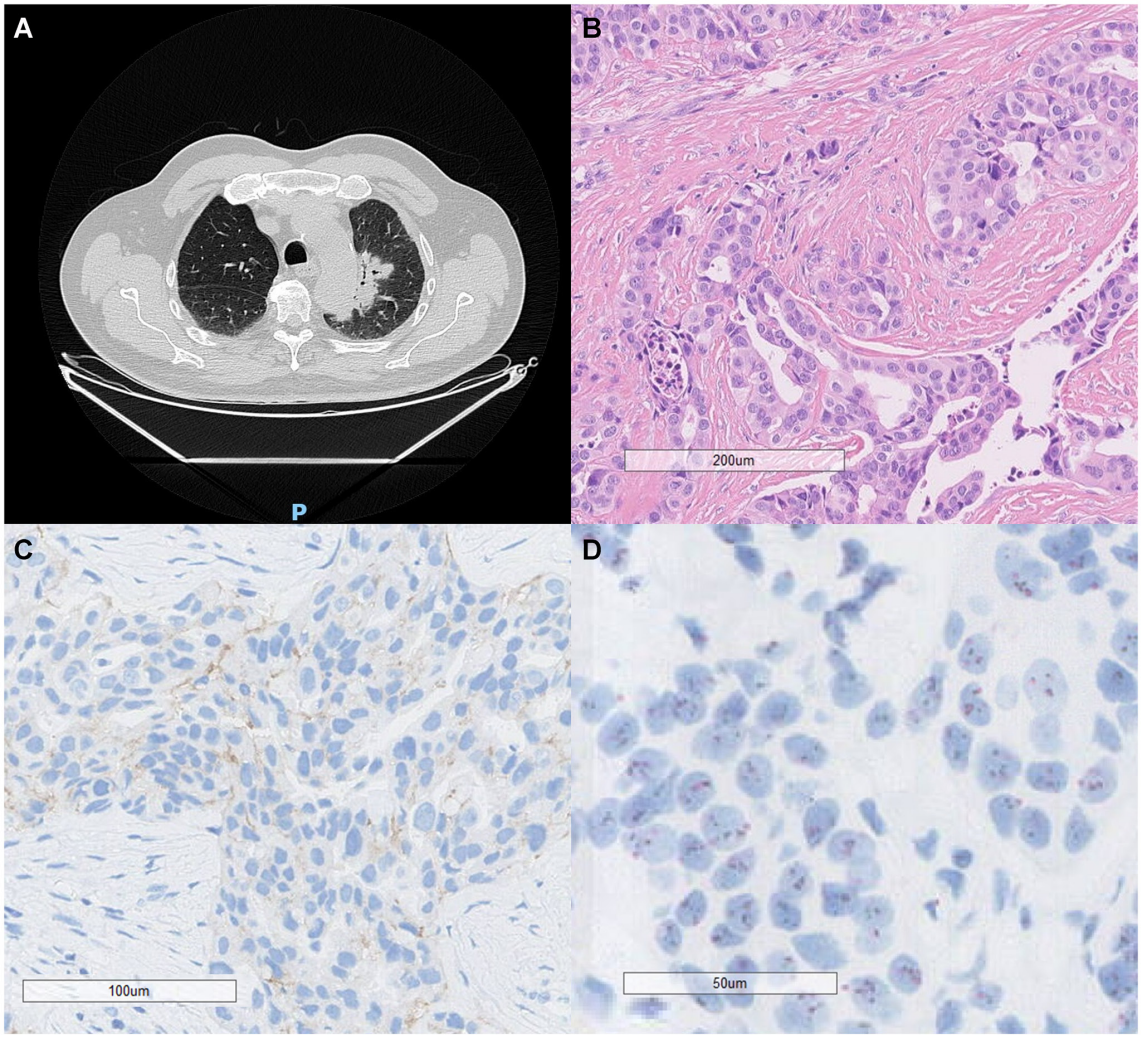
Radiological, histopathological, and molecular features of a HER2-amplified lung adenocarcinoma. (**A**) Chest computed tomography (CT) scan demonstrates a consolidative opacity with air bronchograms, surrounded by a faint ground-glass halo and lobulated margins, located in the visceral periphery of the apicoposterior segment of the left upper lobe. (**B**) Hematoxylin and eosin (H&E) staining reveals acinar-pattern adenocarcinoma with moderately differentiated tumor cells showing glandular architecture (scale bar = 200 μm). (**C**) Immunohistochemistry for HER2 (IHC) demonstrates a 2+ membranous staining pattern, focal basolateral and incomplete, moderate (scale bar = 100 μm). (**D**) Dual *in situ* hybridization (DISH) reveals HER2 gene amplification with an average of 4 HER2 gene copies per centromere (scale bar = 50 μm). These findings confirm the HER2-amplified status of the tumor and support its molecular characterization for targeted therapeutic strategies.

**Figure 4:**
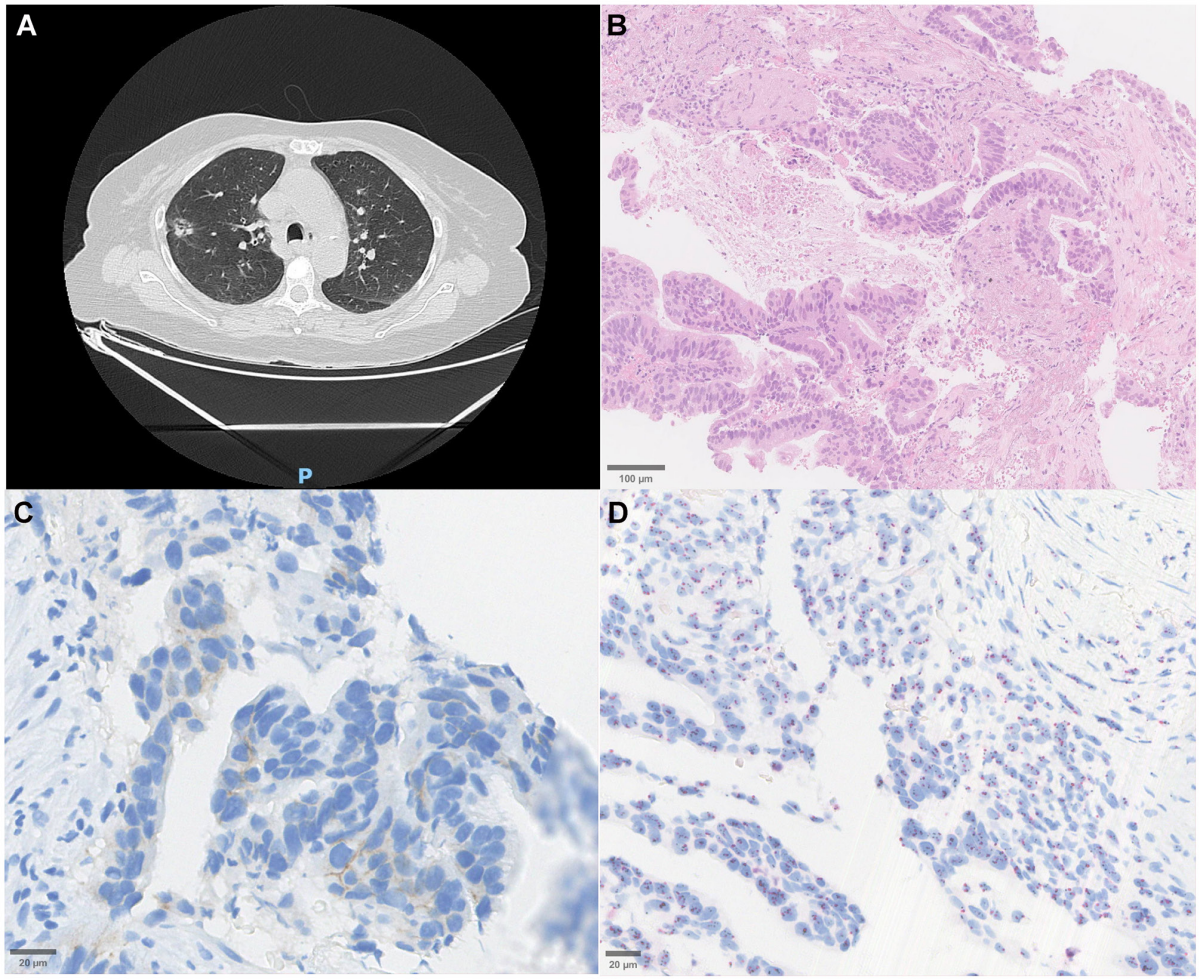
Radiological, histopathological, and molecular features of a HER2 non-amplified lung adenocarcinoma. (**A**) Chest computed tomography (CT) scan reveals a solid nodular lesion with interspersed cystic areas and a subtle ground-glass halo, exhibiting spiculated margins with pleural impressions, located in the anterior segment of the right upper lobe. (**B**) Hematoxylin and eosin (H&E) staining shows a mixed acinar and papillary adenocarcinoma pattern, characterized by glandular and papillary structures lined by atypical epithelial cells (scale bar = 100 μm). (**C**) Immunohistochemistry for HER2 (IHC) demonstrates 1+ membranous staining, indicative of low HER2 expression., with weak incomplete staining in 10% of the tumor (scale bar = 20 μm). (**D**) Dual *in situ* hybridization (DISH) indicates a non-amplified HER2 gene status wi (scale bar = 20 μm).

HER2 protein expression was assessed by immunohistochemistry (IHC) and most cases showed scores 0 or 1+ (8 patients), 2 patients score 2+ and 1 patient, score 3+ (positive), using the protocol for gastric HER2 testing [10]. The HER2 IHC positive case showed DDISH amplification with a 4.2 ratio of HER2 signals to chromosome 17 centromere (Figures 3 and 4). This was the same case that showed amplification in the NGS test (patient #13). None of the cases of IHC 2+ showed amplification on DDISH.

A variety of HER2 mutations were identified, with the most frequent alteration being the A775_G776insYVMA mutation, detected in five patients (38%). Other mutations included G778_P780dup, V842I, L755P, and Q709L. Most mutations were located within exon 20, consistent with previously reported hotspots in HER2-mutated lung cancers. These mutations were identified using next-generation sequencing (NGS), with platforms including Oncomine ThermoFisher and FoundationOne. Co-mutations were common, with TP53 alterations being the most frequent, identified in eight cases (62%). Other recurrent mutations included KRAS, EGFR, STK11, and loss of CDKN2A/B. Notably, some tumors harbored complex genetic landscapes, with multiple concurrent alterations involving genes such as ARID1A, MCL1, MTAP, and ATR. These findings underscore the diverse molecular profiles and potential implications for personalized therapeutic strategies. Tumor mutation burden was available in 9 patients, and were invariably low, ranging from 1 to 13 mutations per megabase.

The heterogeneity of both histological subtypes and molecular alterations in this cohort highlights the complexity of HER2-mutated lung tumors. These data emphasize the importance of comprehensive molecular profiling to guide treatment decisions and to identify potential targets for precision medicine in this challenging subset of lung cancers.

## DISCUSSION

The role of HER2 mutations in lung cancer, particularly non-small cell lung cancer (NSCLC), has garnered significant attention in recent years due to its implications for targeted therapies. The DESTINY-Lung01 Trial demonstrated promising results for the use of trastuzumab deruxtecan (T-DXd) in treating patients with HER2-mutant non-small cell lung cancer (NSCLC). The trial enrolled 91 participants with HER2-mutant NSCLC who had received prior anticancer treatments and showed that 55% of participants responded positively to T-DXd treatment, with a median duration of response lasting 9.3 months, emphasizing the importance of HER2 mutation testing in lung cancer patients to identify those who may benefit from this targeted therapy [6].

This study highlights the clinical, pathological, and molecular complexity of HER2-mutated non-small-cell lung cancer (NSCLC) in a Brazilian cohort, providing valuable insights into a subset of tumors that pose significant diagnostic and therapeutic challenges. The demographic and clinical profiles of our patients reflect patterns previously reported in HER2-mutated NSCLC. A median age of 58 years and a slight female predominance are consistent with prior studies showing a higher prevalence of HER2 mutations in younger, non-smoking or light-smoking populations [11–14]. The cohort’s diversity in smoking history, from never smokers to moderate-to-heavy smokers, suggests that HER2 mutations may be less closely associated with smoking than mutations such as KRAS, but more heterogeneous in their clinical manifestations [13, 15–17].

Recent studies have highlighted that the most prevalent alterations are found in exon 20, with specific mutations such as A775_G776insYVMA being particularly common [18, 19]. These mutations are associated with distinct clinical characteristics; patients often tend to be younger, predominantly female, and have a history of non-smoking [19]. Our study, albeit with a small population, is concordant with these data, as the majority of our patients show this specific genetic variation.

The prevalence of HER2 mutations in our cohort (4.9%) aligns with prior reports from Hong et al. and Arcila et al., who documented a prevalence of 5.2–6% in large-scale retrospective cohorts from the United States and China [20, 21]. Similarly, Parra-Medina et al. described a 4% prevalence of HER2 mutation in a systemic review from papers from Hispanic/Latino population [22]. Another study showed a prevalence of HER2 mutation in 3.2% of the samples from Brazil [23]. The predominant HER2 mutation in our cohort was the exon 20 A775_G776insYVMA insertion. This is in line with the results from Hong et al. and Arcila et al., who reported YVMA insertions in 58–83% of HER2-mutated NSCL [21, 24]. Notably, our study also identified rare HER2 mutations, including G776delins variants, which have been described in these studies, though at a lower frequency.

Our findings highlight the presence of co-mutations in TP53, KRAS, and STK11 in a subset of HER2-mutant tumors. This is consistent with Fang et al., who identified TP53 as the most frequently co-mutated gene in 52% of HER2-mutant cases, with additional alterations in the PI3K/AKT/mTOR pathway contributing to probable resistance to HER2-directed therapies [25]. Hong et al. reported that HER2 alterations were frequently accompanied by EGFR exon 20 insertions, further complicating the genomic landscape [20]. The presence of these co-mutations underscores the need for comprehensive molecular profiling to tailor therapeutic strategies effectively.

HER2-targeted therapies, particularly trastuzumab deruxtecan (T-DXd), have emerged as promising treatment options. In our cohort, we could not assess response rates to HER2-targeted therapies since very few patients had access to this newly approved drug. The findings of Pillai et al. reported a median survival of 2.1 years for HER2-mutant patients receiving targeted therapies compared to 1.4 years in untreated patients [24]. Additionally, Fang et al. demonstrated that specific HER2 variants, such as G778_P780dup and G776delinsVC, derived greater benefit from afatinib than the predominant A775_G776insYVMA variant [25]. Our findings support current guidelines emphasizing that HER2 mutations, particularly exon 20 insertions, represent the most clinically actionable biomarkers for NSCLC, with IHC and DISH serving as supplementary assessments. Importantly, the recent approval of trastuzumab deruxtecan for HER2-mutant NSCLC was primarily based on mutational status rather than protein expression levels, but new emerging data may suggest response to HER2 expressed tumors.

Our cohort showed a higher proportion of HER2 point mutations compared to previous studies, which predominantly reported exon 20 insertions. This discrepancy may be attributed to differences in population genetics, sample selection criteria, or advancements in sequencing methodologies allowing better detection of rare mutations. Point mutations at critical residues such as S310F, V659E, Q709L, L755S, G776C, and D842V exert oncogenic effects by promoting ERBB2 dimerization, stabilizing an active conformation, or increasing kinase activity [26, 27]. Unlike exon 20 insertions, which predominantly activate downstream signaling via steric hindrance in the kinase domain, point mutations often enhance ligand-independent receptor activation, leading to constitutive ERBB2 signaling. Notably, mutations in the extracellular and transmembrane domains, such as S310F and V659E, may confer unique susceptibilities to antibody-drug conjugates targeting HER2, warranting further investigation into their therapeutic implications [20, 26, 27].

The heterogeneity of HER2 alterations observed in our cohort highlights important considerations for testing strategies in clinical practice. While NGS remains the gold standard for comprehensive molecular profiling, its limited availability in many Brazilian healthcare settings necessitates a strategic approach to biomarker testing. Our findings suggest that initial screening with diagnostic immunohistochemistry for lung adenocarcinomas, followed by targeted PCR-based assays for common HER2 exon 20 insertions in non-smoking patients with wild-type EGFR/ALK status, could represent a cost-effective algorithm. This tiered approach could identify most patients who would benefit from HER2-directed therapies while reserving comprehensive NGS for cases with strong clinical suspicion but negative targeted testing. Furthermore, the integration of liquid biopsy into clinical practice could enhance detection rates, particularly in patients with insufficient tissue samples or those requiring longitudinal monitoring for resistance mechanisms.

The concordance between our findings and global data suggests that international clinical trial results for HER2-targeted therapies should be applicable to Brazilian patients, despite potential differences in genetic background or healthcare access. Based on our results, we propose that regional cancer centers establish multidisciplinary molecular tumor boards to optimize treatment selection for HER2-altered NSCLC patients. Such boards could facilitate access to trastuzumab deruxtecan through patient assistance programs or clinical trials while coordinating biospecimen collection for further research into population-specific response determinants. Our identification of patients with concurrent driver mutations also emphasizes the need for novel combination approaches addressing multiple oncogenic pathways simultaneously. Future clinical trials should stratify patients by specific HER2 mutation type and co-mutation status to develop more precise treatment algorithms that account for the molecular complexity revealed in our study.

Some mutations in our dataset were classified as “variants of unknown significance” (VUS), particularly those with limited functional characterization. These variants were carefully evaluated against existing genomic databases and computational models to determine their potential oncogenic relevance. Exclusion of non-pathogenic variants was based on available evidence from preclinical models and large-scale genomic studies, ensuring that only clinically relevant alterations were analyzed. Given the emerging clinical evidence supporting trastuzumab deruxtecan (T-DXd) efficacy in non-traditional HER2 alterations, including point mutations, further clinical validation is required. Reports suggest that T-DXd may be effective against certain extracellular and transmembrane domain mutations, such as S310F and V659E, due to its potent bystander effect and ability to target HER2-expressing tumor cells regardless of mutation type [20, 27].

The molecular landscape of HER2 alterations reveals that these mutations can co-occur with other oncogenic drivers such as TP53 and EGFR mutations, complicating the treatment landscape [18]. Notably, patients with HER2 mutations generally exhibit a lower tumor mutational burden (TMB), which may influence their response to immunotherapies and chemotherapy [28]. The introduction of targeted therapies, including tyrosine kinase inhibitors (TKIs) and antibody-drug conjugates like trastuzumab deruxtecan, has transformed treatment options for HER2-mutant NSCLC. These therapies have shown promising efficacy in clinical trials, particularly for patients with advanced disease [19, 29, 30]. The available data in our cohort of patients with TMB showed that all of them had low TMB, and the majority showed additional mutations to the HER2 gene.

The understanding of HER2’s role extends beyond its mutations to include mechanisms such as hyper-phosphorylation and its impact on tumor biology. Elevated levels of phosphorylated HER2 have been linked to poorer prognoses in some studies, emphasizing the need for comprehensive molecular profiling in NSCLC management [31].

Many studies classify scores of 2+ and 3+ as positive, irrespective of fluorescence *in situ* hybridization (FISH) results, leading to a higher frequency of reported overexpression in lung cancer [12, 28, 32, 33–35]. However, when only a score of 3+ is considered positive, the reported rates become comparable to those observed in this study [15, 35]. HER-2 amplification is relatively rare in NSCLC, with reported frequencies ranging from 0.9 to 14.3% depending on the study and the methods used for detection [35–37]. Odintsov et al. found that high-level HER2 amplification (defined as 6 estimated gene copy ratio) predicted wild-type HER2 overexpression, but in our small series, only one mutated case had HER2 amplification, with a relatively low number of copies of 4 [37]. This case was of interest, since it did not show strong expression by immunohistochemistry, and was classified as 2+ following the gastric HER2 guidelines [38]. In fact, none of our cases showed 3+ positivity, underscoring the need to evaluate both somatic mutations and protein expression by immunohistochemistry in all cases of non-small lung cancer. Takezawa et al. have previously identified HER2 amplification by ISH in three of 26 EGFR mutant cases of lung adenocarcinoma, who progressed on gefitinib or erlotinib [39]. The presence of HER2 amplification in cases with other drives in untreated patients is a subject less well studied.

The molecular complexity in our cohort further supports the need for comprehensive profiling. Tumors often harbored low tumor mutational burden (TMB), ranging from 1 to 13 mutations per megabase, potentially contributing to limited immunogenicity and suboptimal responses to immunotherapy. A meta-analysis by Zhang et al. [40] reported an objective response rate (ORR) of 26% in HER2-mutated NSCLC patients treated with immune checkpoint inhibitor. These rates are relatively modest, indicating limited efficacy of ICIs as monotherapy in this population. However, the combination of ICIs with chemotherapy showed improved outcomes, suggesting that monotherapy may not be sufficient [40]. In a study by Tu et al., [41] patients with non-ex20ins mutations demonstrated superior progression-free survival (PFS) and overall survival (OS) compared to those with ex20ins mutations, suggesting that the specific mutation type significantly impacts immunotherapy efficacy [41]. Tian et al., characterized the genomic and immunogenomic features of lung adenocarcinoma patients with ERBB2 exon 20 insertions. The study highlighted that these patients typically have a low tumor mutational burden (TMB) and are often PD-L1 negative, both of which are associated with a reduced likelihood of responding to immunotherapy with findings similar to our limited cohort [42].

Despite these insights, our study is limited by its retrospective design and small sample size. The cohort’s geographic specificity may also limit the generalizability of findings to broader populations. Future prospective studies with larger sample sizes are needed to validate these results and explore the full clinical implications of HER2-targeted therapies and co-mutation landscapes.

Our study is limited by its small sample size, which constrains the generalizability of our findings. As a result, our conclusions should be interpreted as exploratory rather than definitive. Additionally, given the heterogeneity of HER2 alterations and their clinical implications, larger, multi-institutional studies in Latin America are necessary to validate our results. Such studies would help refine the molecular characterization of HER2-mutant NSCLC and generate hypotheses for further research, particularly regarding treatment responses and biomarker-driven therapeutic strategies.

In conclusion, this study provides a comprehensive overview of HER2-mutated NSCLC within a Brazilian cohort, emphasizing its clinical and molecular heterogeneity. The findings underscore the importance of integrating molecular diagnostics and targeted therapies into clinical practice. A larger, multi-institutional study is needed to comprehensively assess the prevalence and clinical significance of HER2 abnormalities in NSCLC within Brazil. Additionally, a more detailed analysis of the molecular associations of HER2 alterations, including their impact on treatment response and survival, would enhance our understanding of HER2’s role in NSCLC. Such research would contribute significantly to the development of effective HER2-targeted therapies for NSCLC patients.

## MATERIALS AND METHODS

### Participants

We undertook a retrospective study from tissue specimens from 13 patients diagnosed with non-small cell lung cancer at a regional reference laboratory in Northeastern Brazil, with detected HER-2 somatic mutations. Each tissue sample had been previously preserved in 10% buffered formaldehyde, embedded in paraffin, and subsequently sectioned into 5 μm slices, which were stained using the standard hematoxylin and eosin (HE) technique and diagnostic immunohistochemistry (see below). The classification and grading of lung cancers were performed according to the internationally recognized standards set forth by the World Health Organization. This small cohort was part of a series of 263 patients diagnosed with NSCLC and with available NGS panel from 2018–2024. The 13 cases were consecutively identified from a total of 263 NSCLC patients who had NGS testing performed during this period, resulting in a 4.9% prevalence rate. No selection criteria were applied beyond the requirement for histologically confirmed NSCLC and the availability of adequate tissue for molecular analysis, ensuring this cohort is representative of the broader HER2-mutated NSCLC population in our region. The Sankey plot, generated using the ‘SankeyMATIC’ package in R, illustrates the distribution and relationship between case categories.

### Diagnostic immunohistochemistry

Each tumor had been previously formalin-fixed, paraffin-embedded and sectioned at 3–4 μm. A hematoxylin and eosin (HE) staining was performed. Slides were stained with anti-TTF-1 specific monoclonal antibody (prediluted; clone 8G7G3/1; cat. no. 790-4398; Ventana Medical Systems, Inc). We used the Ultraview DAB IHC Detection Kit (cat. no. 760–500; Ventana Medical Systems, Inc.), which includes a blocking reagent, and a secondary antibody conjugated with polymer. Staining was performed using standard automated immunostaining equipment according to the manufacturer’s protocol. IHC slides had a positive control tissue of thyroid tissue for TTF-1. Positive and negative control slides were included in each assay. The slides were re-analyzed by optical microscopy to evaluate the positive and negative controls. While we utilized gastric cancer guidelines for HER2 IHC assessment due to the absence of lung-specific standardized criteria, we acknowledge that HER2 mutations are the primary determinant for HER2-targeted therapy selection in NSCLC, with protein expression and amplification playing supportive roles. This aligns with emerging consensus guidelines from ESMO and IASLC, which prioritize mutational status for therapeutic decision-making in this disease context. This comprehensive molecular profiling approach reflects this understanding, using IHC as a complementary rather than determinative assessment.

### HER-2/neu assays

The assessment of HER-2/neu expression was conducted using both immunohistochemical (IHC) and *in situ* hybridization methods, both using the Roche Ventana Systems/VENTANA BenchMark ULTRA platform (Ventana Medical Systems Inc., Tucson, AZ, USA). In summary, sections measuring 3 to 4 micrometers in thickness were cut from the tissue blocks and placed on pre-treated glass slides. These slides were then dried overnight at 37°C and subsequently at 60°C for 30 minutes, followed by dewaxing and rehydration through a series of graded alcohols. To inhibit endogenous peroxidase activity, the slides were treated with 3% hydrogen peroxide in methanol for 30 minutes. The HER-2/neu status was then evaluated using a monoclonal antibody, anti-HER-2/neu (clone 4B5). HER2 gene amplification was evaluated using the INFORM HER2 Dual *in situ* Hybridization (DISH) DNA Probe Cocktail assay. DNA denaturation was achieved by heating the slides at 80°C. The slides were then incubated with the VENTANA Silver ISH DNP Detection Kit to visualize HER2 copies (indicated by black color), followed by incubation with the VENTANA Red ISH DIG Detection Kit for chromosome 17 (indicated by red color). Finally, the slides were counterstained with hematoxylin.

### HER-2 scoring

HER2 staining was assessed by evaluating membrane expression in tumor cells. According to the 2018 ASCO/CAP guidelines, cases were categorized as negative (score 0 or 1+), equivocal (2+), or positive (3+). Due to the absence of validated scoring systems specific to NSCLC, criteria established for gastric cancer were applied, in line with recommendations by Ren et al. [28]. FT and CN conducted blind evaluations and provided the scores. The ISH results were evaluated based on the ratio of HER2 signals to CEP17 signals and the average HER2 copies in the cancer cells, following the criteria set by ASCO/CAP [38]. Amplification was defined as a HER2/CEP17 ratio ≥2.0, or a ratio <2.0 with ≥6.0 HER2 signals per cell. Equivocal cases had a ratio <2.0 and 4.0–5.9 HER2 signals per cell. Non-amplified cases showed a ratio <2.0 with <4.0 signals per cell.

### HER-2 DDISh computer-assisted validation

All ISH-stained slides were scanned at 40× magnification using the VENTANA DP 200 slide scanner (Roche Diagnostics International, Rotkreuz, Switzerland). The uPath HER2 Dual ISH Image Analysis algorithm was employed to assist interpretation by automating tumor cell detection, signal counting, and HER2/CEP17 ratio calculation. This semi-automated digital tool is designed to mirror the manual evaluation performed by pathologists, as described in prior studies [43].

### Next-generation sequencing

Targeted sequencing was performed in-house using the Ion Torrent^™^ Genexus^™^ Integrated Sequencer (Thermo Fisher Scientific, Waltham, MA, USA) and the corresponding Oncomine Precision Assay (OPA). This fully automated NGS platform integrates library preparation, template generation, sequencing, and data analysis. Variant calling and fusion detection were carried out using Ion Reporter software with custom BED files and appropriate filters. All detected variants were verified using the Integrative Genomics Viewer (IGV v2.3 or higher) to confirm sequence accuracy and exclude artifacts. Data were aligned to the hg19 reference genome, and results were interpreted using the Oncomine^™^ Knowledgebase Reporter. A minimum allele frequency of 5% and median sequencing depth above 500× were used as quality thresholds. Foundation Medicine^®^ assays are comprehensive genomic profiling (CGP) platforms based on next-generation sequencing (NGS), designed to identify somatic alterations—including SNVs, indels, copy number variations (CNVs), and gene rearrangements—across 324 cancer-related genes. These tests also report tumor mutational burden (TMB) and microsatellite instability (MSI) status. TMB was classified as high (≥10 mutations/Mb) or low (<10 mutations/Mb), and MSI as stable or unstable. Testing was conducted through pharmaceutical-sponsored programs, with results accessed via patient medical records.
